# Coronavirus awareness, prevention, and household hardship survey data for the CHAMPS HDSS network: Data collected between August and September of 2022 from the Bamako HDSS, Mali

**DOI:** 10.1016/j.dib.2024.110651

**Published:** 2024-06-19

**Authors:** Jonathan A. Muir, Uduma U. Onwuchekwa, Zachary J. Madewell, Moussa O. Traore, Moussa Kourouma, Fatima Keiri, Solveig A. Cunningham, Samba O. Sow, Milagritos D. Tapia, Karen L. Kotloff

**Affiliations:** a Emory University, Atlanta, GA, United States; b Le Centre pour le Développement des Vaccins du Mali (CVD-Mali), Bamako, Mali; c Centers for Disease Control and Prevention, Atlanta, GA, United States; d Center for Vaccine Development, University of Maryland School of Medicine, Baltimore, MD, United States

**Keywords:** Mali, Resilience, SARS-CoV-2, Vulnerability, West Africa

## Abstract

Data were gathered through a collaborative initiative to investigate impacts of the COVID-19 pandemic and related lockdowns on child and maternal health, economic hardships, and access to care for children and pregnant women by the Child Health and Mortality Prevention Surveillance (CHAMPS) Network. The data were gathered in Bamako, the capital city of Mali (population ∼2.9 million) between August and September of 2022 through a Health and Demographic Surveillance System (HDSS). Data collectors used a survey instrument specifically designed to measure household awareness, knowledge, and prevalence of COVID-19, as well as hardships that households experienced since the onset of the pandemic in March of 2020. The data are from two neighborhoods of Bamako, Banconi and Djicoroni; the Health and Demographic Surveillance System (HDSS) operating in these neighborhoods tracks the health of approximately 235,000 inhabitants. The data were collected using a stratified random sample of 454 households.

Specifications Table

**Table:** 

Subject	Public Health and Health Policy
Specific subject area	Household awareness of COVID-19 and means to prevent disease spread; household experiences of economic and social hardships during the pandemic
Data format	Raw
Type of data	Table
Data collection	Data collection utilized the survey instrument “Harmonized COVID-19 Impact Questions for the CHAMPS HDSS Network”, which was adapted from a standardized instrument. The adapted standardized instruments and a corresponding codebook (data dictionary) are available through the CHAMPS Population Surveillance data repository hosted by *Dataverse* [1]. A detailed study protocol was developed by the coauthors and approved by Le Comité d'Éthique de l'USTTB, approval reference number N^o^0007, and followed during data collection. The data were collected from two predominantly urban communities in Bamako, which is capital city of Mali [2]. The HDSS was established in 2006 by Le Centre pour le Développement des Vaccins du Mali (CVD-Mali). More information about the Bamako HDSS has been published elsewhere [2]. Demographic and health-related information are gathered from the population living within the HDSS during regular rounds of data collection that typically occur one to two times per calendar year. Demographic and health-related data that are regularly collected by the HDSS are distinct from the data collected on COVID-19 impacts for households that are described in this article. All the households within the Bamako HDSS catchment areas were included in the sampling frame and eligible for selection into the study sample. Survey respondents comprised either heads of households or another household member aged 18 years or older at the time of data collection with sufficient knowledge about the household.
Data source location	• Institution: Le Centre pour le Développement des Vaccins du Mali (CVD-Mali) and Emory University • City/Town/Region: Bamako • Country: Mali
Data accessibility	Repository name: CHAMPS Population Surveillance Dataverse Data identification number: https://doi.org/10.15139/S3/MONHOI Direct URL to data: https://dataverse.unc.edu/dataset.xhtml?persistentId=doi:10.15139/S3/MONHOI Instructions for accessing these data: The data are publicly available through the CHAMPS Population Surveillance Dataverse.

## 1 Value of the Data

• Insights into COVID-19 Awareness: This dataset offers insights into household awareness and knowledge of SARS-CoV-2 and COVID-19 in Mali.
• Understanding Socioeconomic Effects: It provides information on socioeconomic shocks experienced by households in Bamako due to lockdowns and related policies implemented to mitigate disease spread during the pandemic period.
• Implications for Child and Maternal Health: These data elucidate repercussions of the pandemic on child and maternal health beyond the immediate impact of the disease itself, highlighting critical healthcare access issues in a resource-limited setting.
• Community-Level Insights: These data can also be used to understand community-level variations in awareness, socioeconomic impacts, and healthcare access.
• Informed Policy Development: The data can inform policy interventions, aiding public health researchers, policymakers, and aid organizations to better address the unique challenges posed by the COVID-19 pandemic and associated lockdowns in resource-limited settings.
• Enhancing Preparedness for Future Pandemics: Insights derived from these data can inform preparedness strategies for future pandemics.
• Versatile Analysis Possibilities: Researchers can analyze these data independently or in combination with other data from the Bamako HDSS. Additionally, these data could be combined into cross-site analyses alongside comparable data from other CHAMPS HDSS sites [[3], [4], [5]]. These data could also be pooled with data from other studies for investigations that extend outside of the CHAMPS Network after proper data harmonization.

## 2 Data Description

The COVID-19 pandemic-related data collected from the Bamako HDSS were part of a collaborative global health initiative among HDSS sites within the Child Health and Mortality Prevention Surveillance Network (CHAMPS) to assess implications and potential consequences of pandemic-related measure, including lockdowns, social distancing, and related policies aimed at stemming the transmission of COVID-19 [[6], [7], [8], [9], [10], [11], [12]]. Specifically, the data were gathered to increase understanding of how these measures may have contributed to broader household socioeconomic challenges. This includes potential impacts on access to healthcare, food availability, and livelihoods, among households, with particular focus on vulnerable groups such as those with children and pregnant women residing in the Bamako HDSS [2]. Data collection did not include formal ascertainment of COVID-19 cases.

The raw data, data codebook, and survey questionnaire are publicly available through a data repository hosted by Dataverse. The data repository is titled the ``CHAMPS Population Surveillance Dataverse.”

Data from a stratified random sample of 454 households that are publicly available through the repository are located in the file titled *Mali – COVID-19 Survey – CHAMPS*. The data are formatted into sections that correspond to the modules of the survey questionnaire:

■ Household information

• Example variables: head of household's age, educational level, marital status, occupation, and sex.

■ Knowledge of COVID-19

• Examples of variables: awareness of COVID-19, awareness of preventive measures (e.g., mask wearing and social distancing) and governmental initiatives to prevent COVID-19 transmission (movement restrictions, fostering public awareness, and lockdowns).

■ Food security

• Examples of variables: food availability, food affordability, and fears associated with COVID-19 that inhibited acquiring food.

■ COVID-19 related economic shocks

• Examples of variables: job loss, business closure, and agricultural disruptions as well as observing price increases for food.

■ Healthcare services for children and pregnant women

Examples of variables: routine follow-up visits; routine vaccination; routine antenatal care, delivery services, and postnatal care; malaria treatment; HIV treatment; clinical visits for any illness; and services for malnutrition.

Variable names follow the naming convention specified in the data dictionary (see supplementary material). For survey questions where respondents could select multiple options, responses were disaggregated into individual variables in the raw data file, each accompanied by descriptive variable labels for clarity.

The data dictionary/codebook is available for download as a PDF file titled *Data Dictionary*.

■ Provides detailed information about the survey instrument, organized into six columns (e.g., type, name, label, etc.); it represents information contained in the survey that was implemented in the field using tablets.
■ Provides information on the response categories for each question—responses are provided as they were originally programmed into the tablets in both English and French.

The survey questionnaire is titled *COVID CHAMPS Mali Instrument*; it includes the survey instrument that was formatted for paper-based data collection, which was later adapted into an electronic format and translated into French, as presented in the codebook.

## 3 Experimental Design, Materials and Methods

Mali is a large, predominantly arid country that is land-locked and situated in West Africa. It covers an area of over 1241,238 square kilometers (∼479,000 sq. mi) and is home to a population of approximately 21.4 million. The population is predominantly Muslim, with a young age structure (nearly 50 % under 15 years), and high adult illiteracy (64.5 %), high adolescent fertility, and limited access to health care [13]. Mali's development and health indicators rank among the lowest in the world according to the United Nations index. In 2002, the Centre pour le Développement des Vaccins du Mali (CVD-Mali) was established as a partnership between the Mali Ministry of Health and the University of Maryland, USA. CVD-Mali is a legal government entity working under the Ministry of Health. Since its inception, CVD-Mali has been dedicated to describing the epidemiology of infectious diseases, supporting the development of vaccines, and training a team of local researchers.

In 2006, CVD-Mali established an HDSS in two quartiers (neighborhoods) in Bamako. One neighborhood, Banconi, is situated in Commune 1 and is in the eastern part of the city, north of the Niger River, and occupies an area of 6.23 km^2^. In 2022, the population was estimated at 153,490. The second neighborhood is Djikoroni-para; it has a surface area of roughly 5 km^2^. It is situated in Commune 4 and is in the western part of Bamako. The estimated population was 82,422 in 2022. Both neighborhoods are densely populated, and the population largely subsists on small business activity, government employment, foreign remittances, and trade. The Bamako HDSS generally conducts biannual demographic data collection rounds as part of its surveillance.

For this auxiliary study, participants were recruited between August and September of 2022. Study participants comprised a qualifying representative (the household head or an adult household member with good knowledge about the household; the study adopted the definition of households and household members used by the HDSS). All households within the Bamako HDSS were eligible for selection into the study, which was implemented during regular HDSS round visits. We used proportion to population stratified random sampling to identify a random sample of 454 households stratified across the two communities covered by the HDSS. During the HDSS round prior to data collection, approximately 62.5 % of households enumerated within the Bamako HDSS resided in the Banconi catchment area. The other 37.5 % resided in the Djikoroni catchment area. Simple random sampling was executed within the two catchment areas (strata) with the goal of obtaining samples from the two strata that were proportionate to the population of households residing therein. Ultimately, we collected data from 317 households residing in Banconi and 137 households residing in Djikoroni, representing 69.8 % and 30.1 % of the sample respectively and indicating a slight over representation of households from the Banconi catchment area and a slight under representation of households from the Djikoroni catchment area that researchers could account for via the use of survey weights.

These self-standing data on COVID-19 impacts are available for independent analysis or in combination with other data from the Bamako HDSS. Additional HDSS data are available upon request to the Bamako HDSS team at CVD-Mali (https://cvd-mali.org/en/home/). Linking the Bamako HDSS data with the COVID-19 data described herein will require HDSS Household IDs that are available for the COVID-19 impact data as well as the broader HDSS data upon request. Linking the two datasets via HDSS Household IDs will enable researchers to combine the COVID-19 impact data with responses in other datasets for specific households in the Bamako HDSS.

The survey instrument was designed following established methods [14], which included identifying key concepts, framing research questions, conducting literature reviews, and drawing from pre-existing questionnaires, such as the “High-Frequency Mobile Phone Surveys of Households to Assess the Impacts of COVID-19″ standardized survey instrument prepared by the World Bank for assessing direct and indirect socioeconomic consequences of the COVID-19 pandemic [15,16]. A standardized survey instrument was prepared in English for implementation across the CHAMPS network, with adaptations for local contexts. The final questionnaire was organized into five sections: respondent identification, SARS-CoV-2 knowledge and practices; histories of symptoms; contacts with people that have COVID-19 symptoms; household economic impacts of COVID-19; and the impacts on healthcare access for household members (see supplementary materials). The instrument did not seek to ascertain COVID-19 cases. The survey prompted participants to reflect on household experiences since March 2020. Data collection was conducted electronically, with both English and French translations of the instrument presented at the same time.

Communication with study participants was in their preferred language. If a fieldworker could not communicate in the preferred language of the respondent, an interpreter was sought from within the household, or the household was revisited at another time by a fieldworker who could communicate in the preferred language.

Data cleaning adhered to standard procedures for the HDSS [17]. Data collectors and supervisors were trained on the study objectives, data confidentiality, and data collection techniques. Data quality concerns such as data inconsistencies, implausible values, or incomplete data were identified and flagged for reconciliation, which was completed by data collectors through return visits to the household.

## Limitations

These data are specific to the communities covered by the Bamako HDSS—they are not generalizable outside of these communities, a limitation common to studies using data collected from HDSS sites [14]. Given that the data were observational, other limitations include possible recall bias and unmeasured variable bias. The data were collected via a cross-sectional study design; thus, they cannot capture temporal variations or indicate causal relationships among indicators. This limits their usefulness in evaluating whether the type and severity of hardships associated with COVID-19 lockdowns have changed over time [7]. One solution to create longitudinal data capturing temporal variations is by repeated administration of the survey instrument during future rounds of HDSS data collection.

## Ethics Statement

This study was conducted according to the guidelines in the Declaration of Helsinki; all procedures involving research study participants, including digital data collection using tablets that were programmed with the corresponding survey instruments, were approved by Le Comité d'Éthique de l'USTTB, approval reference number N^o^0007 and the University of Maryland, Baltimore Institutional Review Board. Written informed consent was obtained for participants who were able to read and write. For participants who were not literate, the informed consent statement was read and oral informed consent from the participant was obtained, documented, and witnessed. These procedures for obtaining written or oral informed consent were approved by Le Comité d'Éthique de l'USTTB.

## CRediT Author Statement

**J.M., U.U., Z.M., S.S., M.T., K.K., and S.C.:** Project administration. **J.M., U.U., Z.M., A.N., C.S., and S.C.:** Conceptualization. **J.M., U.U., Z.M., A.N., C.S., and S.C.:** Methodology. **J.M., U.U., and M.O.T.:** Data curation. **J.M., U.U., Z.M., F.K., M.T., M.O.T., and M.K.:** Validation. **J.M., U.U., and Z.M.:** Formal analysis. **J.M., U.U., and Z.M.:** Visualization. **J.M.:** Writing – original draft. All authors performed writing – review & editing. **S.S., M.T., K.K., and S.C.:** Supervision.

## Data Availability

COVID-19 Impact Data for the CHAMPS HDSS Network: Data from Bamako, Mali (Original data) (Dataverse).

## Appendix Supplementary materials

Image, application 1
