# Supplementary material for: Coronavirus awareness, prevention, and household hardship survey data for the CHAMPS HDSS network: Data collected between August and September of 2022 from the Bamako HDSS, Mali
Source: Data Brief. 2024 Jun 19;55:110651. doi: 10.1016/j.dib.2024.110651 (PMC11372375; doi:10.1016/j.dib.2024.110651)
Supplement: Supplementary file 1 [file mmc1.pdf]

Data Dictionary Codebook

	#	Variable / Field Name	Field Label<br>*Field Note*	Field Attributes (Field Type, Validation, Choices, Calculations, etc.)
**Instrument: Harmonized COVID-19 impact questions for CHAMPS DSS network** (harmonized_covid19_impact_questions_for_champs_dss) Enabled as survey				
	1	[record_id]	Record ID	text
	2	[dss_respondant]	RESPONDENT NUMBER:	text
	3	[dss_hhid]	Household DSS ID:<br>ID DSS du ménage:	text
	4	[dss_indivdss]	Individual DSS ID:<br>ID DSS de l'Individu:	text
	5	[dss_interviewer]	Interviewer's name:<br>Nom de l'enquêteur:	text, Identifier
	6	[dss_intcode]	Interviewer's code:<br>code de l'enquêteur:	text, Identifier
	7	[dss_intdate]	Interview date:<br>Date de l'Interview:	text (date_dmy)
	8	[dss_intstarttime]	Interview start time:<br>Heure de debut de l'interview:	text (time)
	9	[dss_instruct]	INSTRUCTIONS FOR INTERVIEWER<br>1. INSTRUCTIONS HAVE BEEN PROVIDED IN CAPITAL LETTERS. DO NOT READ THESE INSTRUCTIONS TO RESPONDENTS.<br>2. READ ALL QUESTIONS AS WRITTEN. DO NOT READ RESPONSES UNLESS INSTRUCTIONS EXPLICITLY STATE TO DO SO.<br>3. IF QUESTION INSTRUCTIONS STATE TO READ RESPONSE, DO NOT READ "DON'T KNOW" OR "REFUSED TO RESPOND". CIRCLE THESE ONLY IF THEY ARE THE ANSWER GIVEN BY THE RESPONDENTS.<br>4. INDICATE THE RESPONSE THAT CORRESPONDS TO THE RESPONDENT'S ANSWER.<br>5. FOR OPEN QUESTIONS, ENTER THE RESPONDENT'S ANSWER IN THE SPACE PROVIDED.<br><br>INSTRUCTION POUR L'ENQUETEUR<br>1. LES INSTRUCTIONS ONT ETE FOURNIES EN LETTRES MAJUSCULES.NE PAS LIRE CES INSTRUCTIONS AUX REPONDANTS.<br>2. LIRE TOUTES LES QUESTIONS COMME ECRITES.NE LISEZ PAS LES REPONSES SAUF SI LES INSTRUCTIONS INDIQUENT L'ETAT A FAIRE.<br>3. SI LES INSTRUCTIONS DES QUESTIONS INDIQUENT DE LIRE LA REPONSE, NE PAS LIRE 'NE SAIT PAS" OU "REFUS".ENCERCLER-LES UNIQUEMENT S'ILS SONT LA REPONSE DONNEE PAR LES REPONDANTS.<br>4. INDIQUER LA REPONSE QUI CORRESPOND A LA REPONSE DES REPONDANTS.	descriptive

			5. POUR LA QUESTION OUVERTE, ENTRER LA REPONSE DU REPONDANT SUR L'ESPACE PREVU A CET EFFET.	
	10	[dss_d1]	Section Header: *Household Demographics Données démographiques des ménages INTERVIEWER TO PARTICIPANT: I would like to ask the head of household or their spouse some questions about your household. L'ENQUETEUR AU PARTICIPANT: J'aimerais poser quelques questions au chef de ménage ou a leur épouse concernant le ménage.*<br><br>D1. What is your relationship with the head of household? D1. Quelle est votre relation avec le chef de ménage? (THE RESPONDENT SHOULD BE EITHER THE HEAD OF HOUSEHOLD OR THEIR SPOUSE, BUT ADDITIONAL OPTIONS ARE PROVIDED) (LE REPONDANT DOIT ETRE SOIT LE CHEF DE MENAGE OU SON EPOUSE, MAIS DES OPTIONS SUPPLEMENTAIRES SONT PREVUES)	radio<br>1 Household Head**Chef de ménage<br>2 Spouse of Household Head**Epouse du chef de ménage<br>3 Son**Fils<br>4 Daughter**Fille<br>5 Adopted son**Fils Adoptif<br>6 Adopted daughter**Fille Adoptive<br>7 Nephew**Neveux<br>8 Niece**Nièce<br>9 Grandson**Petit-fils<br>10 Granddaughter**Petite-fille<br>11 Father of Household Head**Père du chef de ménage<br>12 Mother of Household Head**Mère du chef de ménage<br>13 Brother of Household Head**Frère du chef de ménage<br>14 Sister of Household Head**Sœur du chef de ménage<br>15 Unrelated**Aucun lien<br>88 Other**Autre<br>98 Don't Know**Ne sait pas<br>99 Refused to respond**Refus de répondre
	11	[dss_d2]	D2. What is the current age of the head of household? D2. Quel est l'âge actuel du chef de ménage? (NUMBER OF YEARS) (NOMBRE D'ANNEE) ENTER 998 IF "DON'T KNOW".ENTER 999 IF "REFUSED TO ANSWER".	text<br>Custom alignment: RH
	12	[dss_d3]	D3. What is the sex of the head of household?<br><br>D3. Quel est le sexe du chef de ménage?	radio<br>1 Male**Masculin<br>2 Female**Féminin<br>98 Don't know**Ne sait pas<br>99 Refused to respond**Refus de répondre
	13	[dss_d4]	D4. What is the current marital status of the head of household?<br><br>D4. Quel est le statut matrimonial du chef de ménage?	radio<br>1 Married/cohabitating**Marié; Cohabitation<br>2 Divorced**Divorcé<br>3 Single/Never married**Célibataire/Jamais marié<br>4 Widow/Widower**Veuve/Veuf

				5 Separated**Séparé<br>88 Other**Autre<br>98 Don't know**Ne sait pas<br>99 Refused to respond**Refus de répondre
	14	[dss_d5]	D5. How many years of education has the head of household completed?<br><br>D5. Quel est le niveau d'étude du chef de ménage?<br><br>ENTER 98 IF "DON'T KNOW".<br>ENTER 99 IF "REFUSED TO ANSWER".	text<br>Custom alignment: RH
	15	[dss_d6]	D6. Is the head of household currently employed?<br><br>D6. Est-ce que le chef de ménage travaille actuellement?	radio<br>1 Yes**Oui<br>2 No**Non<br>98 Don't know**Ne sait pas<br>99 Refused to respond**Refus de repondre
	16	[dss_d7]<br><br>Show the field ONLY if:<br>[dss_d6] = '1'	D7. What is the head of household's type of occupation?<br><br>D7. Quel est le type d'occupation du chef de ménage?	radio<br>1 Not working**Ne travaille pas<br>2 Professional/Technical/Managerial** Professionnel/Technique/Managérial<br>3 Clerical**Clérical<br>4 Sales**Vente<br>5 Services**Services<br>6 Agricultural - employee**Employé agricole<br>7 Skilled manual labor**Main d'œuvre qualifiée<br>8 Unskilled manual labor**Main d'œuvre non qualifiée<br>88 Other**Autre<br>98 Don't know**Ne sait pas<br>99 Refused to respond**Refus de répondre
	17	[dss_d7other]<br><br>Show the field ONLY if:<br>[dss_d7] = '88'	Other, specify<br><br>Autre, specifier	text
	18	[dss_d8]	D8. What is the religion of the head of household?<br><br>D8. Quelle est la religion du chef de ménage?	radio<br>1 Islam**Islam/musulman<br>2 Christian**Chrétien<br>3 Traditional**Traditionnel<br>4 Atheist**Athée<br>88 Other**Autre<br>98 Don't know**Ne sait pas

				99 Refused to respond**Refus de répondre
	19	[dss_d8other]<br><br>Show the field ONLY if:<br>[dss_d8] = '88'	Other, specify<br><br>Autre, specifier	text<br>Custom alignment: RH
	20	[dss_d9]	D9. What is the ethnicity of the head of household?<br><br>D9. Quelle est l'ethnie du chef de ménage?	radio<br>1 Bambara**Bambara<br>2 Fula**Fula/Peuhl<br>3 Soninke**Soninke<br>4 Senufo**Senoufo<br>5 Malinke**Malinké<br>6 Dogon**Dogon<br>7 Songhai**Songhaï<br>8 Tuareg**Touareg<br>9 Bobo**Bobo<br>88 Other**Autre<br>98 Don't know**Ne sait pas<br>99 Refused to respond**Refus de répondre
	21	[dss_d9other]<br><br>Show the field ONLY if:<br>[dss_d9] = '88'	Other, specify<br>Autre, specifier	text
	22	[dss_10]	D10. How many people are in your household?<br><br>D10. Combien de personnes Ya-il dans votre ménage?<br><br>(TOTAL COUNT OF HOUSEHOLD MEMBERS)<br><br>(NOMBRE TOTAL DES MEMBRES DU MENAGES)<br><br>ENTER 98 IF "DON'T KNOW".<br>ENTER 99 IF "REFUSED TO ANSWER".	text<br>Custom alignment: RH
	23	[dss_d11]	D11. How many children under the age of 5 are in the household?<br><br>D11. Ya-t-il combien d'enfant de moins de 5 ans dans le ménage?<br><br>ENTER 98 IF "DON'T KNOW".<br>ENTER 99 IF "REFUSED TO ANSWER".	text<br>Custom alignment: RH
	24	[dss_d12]	D12. How many individuals are 60 years old or older in the household?<br><br>D12. Combien de personnes ont 60 ans ou plus dans le ménage?<br><br>ENTER 98 IF "DON'T KNOW".<br>ENTER 99 IF "REFUSED TO ANSWER".	text<br>Custom alignment: RH
	25	[dss_13]	D13. What is the average total monthly income of your household?	text

			D13. Quel est le revenu mensuel de votre ménage?<br><br>(REPORT IN WHOLE UNITS OF LOCAL CURRENCY)<br><br>(RAPPORT EN UNITES ENTIERES DE LA MONNAIES LOCALE)<br><br>ENTER 98 IF "DON'T KNOW".<br>ENTER 99 IF "REFUSED TO ANSWER".	
	26	[dss_v1]	Section Header: *Knowledge Regarding the Spread of COVID-19 Connaissances impliquant la propagation du COVID-19 INTERVIEWER TO PARTICIPANT: I would like to ask the head of the household some questions about coronavirus or COVID-19. L'ENQUETEUR AU PARTICIPANT: J'aimerais vous poser des questions au chef de ménage ou à leur épouse sur le coronavirus ou COVID-19.*<br>V1. Have you heard about the coronavirus/COVID-19?<br><br>V1. Avez-vous entendu parler du coronavirus/COVID-19?	radio<br>1 Yes**Oui<br>2 No**Non<br>98 Don't know**Ne sait pas<br>99 Refused to respond**Refus de repondre
	27	[dss_v2]<br><br>Show the field ONLY if:<br>[dss_v1] = '1'	V2. To your knowledge, which of the following measures can you adopt to reduce the risk of contracting coronavirus/COVID-19?<br><br>V2. A votre connaissance, lesquelles des mesures suivantes pouvez-vous adopter pour réduire le risque de contracter le coronavirus.COVID-19?<br><br>(PLEASE DO NOT READ, CHECK MULTIPLE RESPONSES THAT APPLY, OR WRITE RESPONSE FOR "OTHER" IF SELECTED)<br><br>(Ne lisez pas s'il vous plait, vérifiez les multiples réponses qui sont appliquées, ou écrivez 88 comme réponse si vous ne savez pas)	checkbox<br>1 dss_v2___1 Handwashing**Lavage des mains<br>2 dss_v2___2 Sanitizer Use**Utilisation de désinfectant<br>3 dss_v2___3 Avoid handshake/physical greeting**Eviter la poignée de main/Salutation physique<br>4 dss_v2___4 Mask use**Utilisation du masque<br>5 dss_v2___5 Gloves use**Utilisation des gants<br>6 dss_v2___6 Avoid travel**Eviter les voyages<br>7 dss_v2___7 Staying at home**Rester a la maison<br>8 dss_v2___8 Avoid going out unless necessary**Eviter de sortir à moins que cela ne soit nécessaire<br>9 dss_v2___9 Avoid crowded places**Eviter les places publiques (bondées)<br>10 dss_v2___10 Keep ~2 meters space between yourself and others**Gardez 2 mètres d'espace entre les autres et vous<br>88 dss_v2___88 Other**Autres<br>98 dss_v2___98 Don't know**Ne sait pas

				99 | dss_v2___99 | Refused to respond**Refus de Repondre
	28	[dss_v2handother]<br><br>Show the field ONLY if:<br>[dss_v2(88)] = '1'	Other, specify<br><br>Autre, specifier	text
	29	[dss_v3]<br><br>Show the field ONLY if:<br>[dss_v1] = '1'	V3. What steps has your community/government taken to curb the spread of the coronavirus in your area?<br><br>V3. Quelles sont les mesures prises par votre communauté/gouvernement) pour freiner la propagation du coronavirus dans votre région?<br><br>(PLEASE READ ALOUD, SELECT ALL THAT APPLY)<br><br>(LISEZ A HAUTE VOIX, SELECTIONNEZ TOUT CE QUI S'APPLIQUE)	checkbox<br>0 | dss_v3___0 | None**Aucun<br>1 | dss_v3___1 | Advised citizens to stay at home**Conseiller les citoyen [illegible] la maison<br>2 | dss_v3___2 | Advised to avoid gatherings[illegible] les gens a éviter les rassembl[illegible]<br>3 | dss_v3___3 | Restricted travel within country/area**Restreindre le[illegible] déplacements a l'interieur du pays/Zone<br>4 | dss_v3___4 | Restricted international travel**Restreindre les voyag[illegible] internationaux<br>5 | dss_v3___5 | Closure of schools and universities**Fermeture des [illegible] Universités<br>6 | dss_v3___6 | Curfew/lockdown**Couvre-feu/confinement<br>7 | dss_v3___7 | Closure of non-essential businesses**Fermeture des [illegible] non essentielles<br>8 | dss_v3___8 | Sensitization/public awareness**Sensibilisation/I[illegible] publiques<br>9 | dss_v3___9 | Established isolation centers**Etablissement de ce[illegible] d'isolement<br>10 | dss_v3___10 | Disinfection of public places**Désinfection des pla[illegible] publiques<br>88 | dss_v3___88 | Other**Autre<br>98 | dss_v3___98 | Don't know**Ne sait pas<br>99 | dss_v3___99 | Refused to respond**Refus d[illegible] répondre
	30	[dss_v3other]<br><br>Show the field ONLY if:<br>[dss_v3(88)] = '1'	Other, specify<br><br>Autre, specifier	text
	31	[dss_v4]<br><br>Show the field ONLY if:<br>[dss_v1] = '1'	V4. Were any of your household members tested for COVID-19?<br><br>V4. Est-ce que l'un des membres de votre ménage a-t-il subi un test de dépistage de la COVID-19?	radio<br>1 | Yes**Oui<br>2 | No**Non<br>98 | Don't know**Ne sait pas

				99 Refused to respond**Refus de repondre
	32	[dss_v5]<br><br>Show the field ONLY if:<br>[dss_v4] = '1'	V5. Did any of your household members test positive for COVID-19?<br><br>V5. L'un des membres de votre ménage a-t-il été teste positif au COVID-19?	radio<br>1 Yes**Oui<br>2 No**Non<br>98 Don't know**Ne sait pas<br>99 Refused to respond**Refus de repondre
	33	[dss_p1]	Section Header: *Food availability (P) DISPONIBILITE DE SUBSTANCE NUTRITIVE (P) INTERVIEWER TO PARTICIPANT: Getting enough food can also be a problem for some households due to coronavirus or COVID-19. I would like to ask you some questions about food availability in your household. L'ENQUETEUR AU PARTICIPANT: Obtenir (avoir) assez de nourriture peut aussi être un problème pour certains ménages due au coronavirus ou COVID-19. J'aimerais vous poser quelques questions à propos de la disponibilité de la nourriture dans votre ménage.*<br><br>P1a. Before mid-March 2020, did it ever happen that your household did not have enough food to eat?<br><br>P1a. Avant mi-Mars 2020, est-il arrivé que votre ménage n'ait pas assez de nourriture à manger?	radio<br>1 Yes**Oui<br>2 No**Non<br>98 Don't know**Ne sait pas<br>99 Refused to respond**Refus de repondre
	34	[dss_p1b]<br><br>Show the field ONLY if:<br>[dss_p1] = '1'	P1b. How many times did your household not have enough food to eat?<br><br>P1b. Combien de fois, votre ménage n'a pas eu assez de nourriture à manger?<br><br>ENTER 98 IF "DON'T KNOW".<br>ENTER 99 IF "REFUSED TO ANSWER".	text
	35	[dss_p1c]<br><br>Show the field ONLY if:<br>[dss_p1] = '1'	P1c. Why did your household not have enough to eat or not what you wanted to eat before mid-March 2020?<br><br>P1c. Pourquoi votre ménage n'avait pas assez à manger ou ne (voulait) désirait-Il pas manger avant mi-Mars 2020?<br><br>(SELECT ALL THAT APPLY)<br><br>(SELECTIONNER TOUT CE QUI S'APPLIQUE)	checkbox<br>1 dss_p1c___1 Couldn't afford to buy more food**Je ne pouvais me permettre d'acheter plus de nourriture<br>2 dss_p1c___2 Couldn't get out to buy food**Je ne pouvais pas sortir pour acheter à manger<br>3 dss_p1c___3 Afraid to go or didn't want to go out to buy food**Peur de sortir pour aller acheter de la nourriture<br>4 dss_p1c___4 Couldn't get groceries or meals delivered to me**Ne pouvais pas aller à l epicerie ou me faire livrer des repas<br>5 dss_p1c___5 Stores didn't have the food I wanted**Les magasins n'avaient pas

				la nourriture que je voulais<br>88 | dss_p1c___88 | Other**Autre<br>98 | dss_p1c___98 | Don't know**Ne sait pas<br>99 | dss_p1c___99 | Refused to respond**Refus de répondre
	36	[dss_p2other]<br>Show the field ONLY if:<br>[dss_p1c(88)] = '1'	Other, specify<br><br>Autre, specifier	text
	37	[dss_p2a]	P2a. Since the beginning of the COVID lockdown, has it happened that your household did not have enough food to eat?<br><br>P2. Depuis le confinement de la COVID-19, est-il arrivé que votre ménage n'ait pas assez à manger?	radio<br>1 | Yes**Oui<br>2 | No**Non<br>98 | Don't know**Ne sait pas<br>99 | Refused to respond**Refus de repondre
	38	[dss_p2b]<br>Show the field ONLY if:<br>[dss_p2a] = '1'	P2b. During this time, how many times did your household not have enough food to eat?<br><br>P2b. Pendant cette période, combien de fois votre ménage n'a-t-il pas eu assez de nourriture à manger?<br><br>ENTER 98 IF "DON'T KNOW".<br>ENTER 99 IF "REFUSED TO ANSWER".	text
	39	[dss_p3]<br>Show the field ONLY if:<br>[dss_p2a] = '1'	P3. Why did your household not have enough to eat (or not what you wanted to eat) since mid-March 2020?<br><br>P3. Pourquoi votre ménage n'avait-il pas assez à manger(ou pas que vous vouliez manger) depuis mi-Mars 2020?<br><br>(SELECT ALL THAT APPLY)<br><br>(SELECTIONNER TOUT CE QUI S'APPLIQUE)	checkbox<br>1 | dss_p3___1 | Couldn't afford to buy more food**Je ne pouvais me permettre d'acheter plus de nourriture<br>2 | dss_p3___2 | Couldn't get out to buy food**Je ne pouvais pas sortir pour acheter à manger<br>3 | dss_p3___3 | Afraid to go or didn't want to go out to buy food**Peur de sortir pour aller acheter de la nourriture<br>4 | dss_p3___4 | Couldn't get groceries or meals delivered to me**Ne pouvais pas aller à l epicerie ou me faire livrer des repas<br>5 | dss_p3___5 | Stores didn't have the food I wanted**Les magasins n'avaient pas la nourriture que je voulais<br>88 | dss_p3___88 | Other**Autre<br>98 | dss_p3___98 | Don't know**Ne sait pas

				99 | dss_p3___99 | Refused to respond**Refus de répondre
	40	[dss_p4other]<br><br>Show the field ONLY if:<br>[dss_p3(88)] = '1'	Other, specify<br><br>Autre, specifier	text
	41	[dss_m0]	Section Header: *COVID-19 related shocks/coping (M) Faire face aux chocs lies a la COVID-19 (M) INTERVIEWER TO PARTICIPANT: Now I would like to ask you about events that may have affected your household since mid-March. L'ENQUETEUR AU PARTICIPANT: Je voudrais vous posez des questions maintenant sur les évènements qui ont pu affecter votre ménage depuis mi-Mars.*<br><br>M0. Before mid-March of 2020, were any members of your household engaged in any of the following activities?<br><br>M0. Avant mi-Mars 2020, est- ce que des membres de votre ménage participaient aux activités suivantes?<br><br>(PLEASE READ ALOUD AND SELECT ALL THAT APPLY)<br><br>(VEUILLEZ LIRE A HAUTE VOIX ET SELECTIONNER TOUS CEUX QUI S'APPLIQUENT)	checkbox<br>1 | dss_m0___1 | Employment**Emploi<br>2 | dss_m0___2 | Non-farm business operation**Exploitation entreprise non agricole<br>3 | dss_m0___3 | Farming/Agriculture operation**Agriculture/ Exploitation agricole<br>98 | dss_m0___98 | Don't know**Ne sait pas<br>99 | dss_m0___99 | Refused to respond**Refus de répondre
	42	[dss_m1]	M1. Has your household been affected by any of these events since mid-March?<br><br>M1. Est-ce que votre ménage a-t-il été touché par l'un de ces évènements depuis la mi-Mars?<br><br>(PLEASE READ ALOUD AND SELECT ALL THAT APPLY)<br><br>(VEUILLEZ LIRE A HAUTE VOIX ET SELECTIONNER TOUS CEUX QUI S'APPLIQUENT)	checkbox<br>1 | dss_m1___1 | Job loss**Perte d'emploi<br>2 | dss_m1___2 | Nonfarm business closure**Fermeture d'entreprise non agricole<br>3 | dss_m1___3 | Disruption of farming**Perturbation (interruption) de l'agriculture<br>4 | dss_m1___4 | Disruption of livestock activities**Perturbation (interruption) des activités d'élevage<br>5 | dss_m1___5 | Disruption of fishing activities**Perturbation (interruption) des activités de pêche<br>6 | dss_m1___6 | Increased price of farming or business inputs**Augmentation du prix des intrants agricoles ou commerciaux<br>7 | dss_m1___7 | Decreased price of farming or business outputs**Diminution du prix de l'agriculture ou des productions commerciales<br>8 | dss_m1___8 | Increased price of major food items consumed**Augmentation du prix des principaux

				produits alimentaires consommés<br>9 · dss_m1___9 · Illness, injury, or death of any household member**Maladie, blessure, ou le décès d'un membre du ménage<br>10 · dss_m1___10 · Not affected by any listed or other major problems/events**N'est pas affecté par un autre problème/évènement majeur énuméré<br>88 · dss_m1___88 · Other major problem**Autre<br>98 · dss_m1___98 · Don't know**Ne sait pas<br>99 · dss_m1___99 · Refused to respond**Refus de répondre
	43	[dss_m1other]<br><br>Show the field ONLY if:<br>[dss_m1(88)] = '1'	Other, specify<br><br>Autre, specifier	text
	44	[dss_m2]<br><br>Show the field ONLY if:<br>[dss_m1(1)] = '1' or [dss_m1(2)] = '1' or [dss_m1(3)] = '1' or [dss_m1(4)] = '1' or [dss_m1(5)] = '1' or [dss_m1(6)] = '1' or [dss_m1(7)] = '1' or [dss_m1(8)] = '1' or [dss_m1(9)] = '1' or [dss_m1(88)] = '1' or [dss_m1(98)] = '1'	M2. How did your household cope with these difficulties encountered since mid-March?<br><br>M2. Comment votre ménage a-t-il fait face à ces difficultés rencontrées depuis mi-Mars<br><br>(PLEASE DO NOT READ, SELECT ALL THAT APPLY)<br><br>(NE LISEZ PAS S'IL VOUS PLAIT, SELECTIONNER TOUS CEUX QUI S'APPLIQUENT)	checkbox<br>0 · dss_m2___0 · Did nothing**N'a rien fait<br>1 · dss_m2___1 · Sale of assets**Vente d'actifs<br>2 · dss_m2___2 · Engaged in additional income-generating activities**Engager les Revenus supplémentaires dans les activités produites<br>3 · dss_m2___3 · Assistance from friends & family**Assistance des ami(es) et de la famille<br>4 · dss_m2___4 · Borrowed from friends & family**Emprunter à des amis (es)et à la famille<br>5 · dss_m2___5 · Took a loan**Contracter un prêt<br>6 · dss_m2___6 · Delayed payment obligations**Retard de paiement<br>7 · dss_m2___7 · Sold harvest in advance**Vente de la récolte en avance<br>8 · dss_m2___8 · Reduced food consumption**Réduction de la consommation alimentaire<br>9 · dss_m2___9 · Reduced nonfood consumption**Réduction

						de la consommation non alimentaire<br>10 | dss_m2___10 | Relied on savings**Economisez (Epargner)<br>11 | dss_m2___11 | Received assistance from NGO**Reçu Assistance (aide) d'une ONG<br>12 | dss_m2___12 | Took advanced payment from employer**Recevoir un payement anticipé de l'employeur<br>13 | dss_m2___13 | Received assistance from government**Reçu L'aide du gouvernement<br>88 | dss_m2___88 | Other**Autre<br>98 | dss_m2___98 | Don't know**Ne sait pas<br>99 | dss_m2___99 | Refused to respond**Refus de Répondre
	45	[dss_m2other]<br><br>Show the field ONLY if:<br>[dss_m2(88)] = '1'	Other, specify<br><br>Autre, specifier	text
	46	[dss_q0]	Section Header: *Under-five child healthcare services (Q) Services de soins de santé pour les enfants de moins de 5 ans (Q) INTERVIEWER TO PARTICIPANT: Getting adequate health services can be a problem due to coronavirus or COVID-19. I would like to ask some questions about the children under age 5 years in your household. L'ENQUETEUR AU PARTICIPANT: Obtenir des services de santé adéquats peuvent être un problème du au coronavirus ou COVID-19.J'aimerais vous posez quelques questions sur les enfants de moins de 5 ans de votre ménage.*<br><br>Q0. Since mid-March 2020, were there any children under the age of 5 living in the household?<br><br>Q0. Depuis mi-Mars 2020, y avait-il des enfants de moins de 5 ans vivant dans le ménage?	radio<br>1 | Yes**Oui<br>2 | No**Non<br>98 | Don't know**Ne sait pas<br>99 | Refused to respond**Refus de repondre
	47	[dss_q1]<br><br>Show the field ONLY if:<br>[dss_q0] = '1'	Q1. Since mid-March 2020, did any of the children under age 5 attend any healthcare visits?<br><br>Q1. Depuis mi-Mars, est-ce que l'un des enfants de moins de 5 ans a-t-Il assisté à des visites de soins de santé?	radio<br>1 | Yes**Oui<br>2 | No**Non<br>98 | Don't know**Ne sait pas<br>99 | Refused to respond**Refus de repondre
	48	[dss_q2]<br><br>Show the field ONLY if:<br>[dss_q1] = '1'	Q2. What kind of medical care did the child receive?<br><br>Q2. Quel type de soins médicaux l'enfant a-t-il reçu?<br><br>(PLEASE DO NOT READ, SELECT ALL THAT APPLY)	checkbox<br>1 | dss_q2___1 | Routine follow-up visits for kids**Visites de Suivi de routine pour les enfants<br>2 | dss_q2___2 | Routine vaccinations**Vaccinations de routine

			(VEUILLEZ LIRE A HAUTE VOIX, SELECTIONNER TOUS CEUX QUI S'APPLIQUENT)	3 dss_q2___3 Malaria treatment**Traitement du paludisme<br>4 dss_q2___4 HIV treatment**Traitement du VIH<br>5 dss_q2___5 Clinic visits for any illness**Visites à la clinique pour toutes maladies<br>6 dss_q2___6 Services for malnutrition**Services pour la malnutrition<br>88 dss_q2___88 Other**Autre<br>98 dss_q2___98 Don't know**Ne sait pas<br>99 dss_q2___99 Refused to respond**Refus de répondre
	49	[dss_q2other]<br><br>Show the field ONLY if:<br>[dss_q2(88)] = '1'	Other, specify<br><br>Autre, specifier	text
	50	[dss_q3]	Q3. Since mid-March, was there a time you needed medical care or clinic visit for a baby or child but could not do so?<br><br>Q3. Depuis mi-Mars, y a-t-il eu un moment ou vous avez eu besoin de soins médicaux ou d'une visite à la clinique pour un bébé ou un enfant mais que n'avez pas pu faire?	radio<br>1 Yes**Oui<br>2 No**Non<br>98 Don't know**Ne sait pas<br>99 Refused to respond**Refus de repondre
	51	[dss_q3a]<br><br>Show the field ONLY if:<br>[dss_q3]='1'	Q3a. During this time, how many medical care or clinical visits were missed?<br><br>Q3a. Pendant cette période, combien de soins médicaux ou de visites médicales ont été manquées?	text
	52	[dss_q4]<br><br>Show the field ONLY if:<br>[dss_q3] = '1'	Q4. What kind of medical care did the child need but did not receive?<br><br>Q4. Quel type de soins l'enfant avait-il besoin mais n'a pas reçu?<br><br>(PLEASE DO NOT READ, SELECT ALL THAT APPLY)<br><br>(NE LISEZ PAS S'IL VOUS PLAIT, SELECTIONNER TOUS CEUX QUI S'APPLIQUENT)	checkbox<br>1 dss_q4___1 Routine follow-up visits for kids**Visites de suivi de routine pour les enfants<br>2 dss_q4___2 Routine vaccinations**Vaccinations de routine<br>3 dss_q4___3 Malaria treatment**Traitement du paludisme<br>4 dss_q4___4 HIV treatment**Traitement du VIH<br>5 dss_q4___5 Clinic visits for any illness**Visites cliniques pour toutes maladies<br>6 dss_q4___6 Services for malnutrition**Services

				<table><tr><td></td><td></td><td>pour la malnutrition</td></tr><tr><td>88</td><td>dss_q4___88</td><td>Other**Autre</td></tr><tr><td>98</td><td>dss_q4___98</td><td>Don't know**Ne sait pas</td></tr><tr><td>99</td><td>dss_q4___99</td><td>Refused to respond**Refus de répondre</td></tr></table>
	53	[dss_q4other]<br><br>Show the field ONLY if:<br>[dss_q4(88)] = '1'	Other, specify<br><br>Autre, specifier	text
	54	[dss_q5]<br><br>Show the field ONLY if:<br>[dss_q3] = '1'	Q5. Why did your child not receive healthcare?<br><br>Q5. Pourquoi votre enfant n'a pas reçu les soins de santé?<br><br>(PLEASE DO NOT READ, SELECT ALL THAT APPLY)<br><br>(NE LISEZ PAS S'IL VOUS PLAIT SELECTIONNER TOUS CEUX QUI S'APPLIQUENT)	checkbox<table><tr><td>1</td><td>dss_q5___1</td><td>Clinic closed**Fermeture de la clinique</td></tr><tr><td>2</td><td>dss_q5___2</td><td>Out of vaccines or medications**A court de vaccin ou de médicaments</td></tr><tr><td>3</td><td>dss_q5___3</td><td>Did not get transportation**Faute de moyen de transport</td></tr><tr><td>4</td><td>dss_q5___4</td><td>Lockdown**Confinement</td></tr><tr><td>5</td><td>dss_q5___5</td><td>Scared to go**Peur d'y aller</td></tr><tr><td>88</td><td>dss_q5___88</td><td>Other**Autre</td></tr><tr><td>98</td><td>dss_q5___98</td><td>Don't know**Ne sait pas</td></tr><tr><td>99</td><td>dss_q5___99</td><td>Refused to respond**Refus de répondre</td></tr></table>
	55	[dss_q5other]<br><br>Show the field ONLY if:<br>[dss_q5(88)] = '1'	Other, specify<br><br>Autre, specifier	text
	56	[dss_r]	Section Header: *Healthcare services for pregnant women (R) Services de Soins de Santé pour Femme Enceinte(R) L'ENQUETEUR AU PARTICIPANT: Obtenir des services de sante adéquats peuvent être un problème du au coronavirus ou COVID-19.J'aimerais vous posez quelques questions sur les services de santé de la grossesse pendant cette période.*<br>R. Is there a woman in the household who has been pregnant since mid-March 2020?<br><br>R. Y a-t-il eu une femme qui est tombée enceinte depuis mi-Mars 2020?	radio<table><tr><td>1</td><td>Yes**Oui</td></tr><tr><td>2</td><td>No**Non</td></tr></table>
	57	[dss_rinstruct]<br><br>Show the field ONLY if:<br>[dss_r] = '1'	I would like to ask about the pregnancy health services received during this time.	descriptive
	58	[dss_r1]<br><br>Show the field ONLY if:<br>[dss_r] = '1'	R1. Since mid-March, did you attend any pregnancy-related healthcare?<br><br>R1. Depuis mi-Mars, avez-vous assisté à des soins de santé lies à la grossesse?	radio<table><tr><td>1</td><td>Yes**Oui</td></tr><tr><td>2</td><td>No**Non</td></tr><tr><td>98</td><td>Don't know**Ne sait pas</td></tr><tr><td>99</td><td>Refused to respond**Refus de repondre</td></tr></table>

	59	[dss_r2]<br><br>Show the field ONLY if:<br>[dss_r1] = '1'	R2. What kind of healthcare during pregnancy was received since mid-March?<br><br>R2. Quel type de soins de santé a été reçu pendant la grossesse depuis mi-Mars?<br><br>(PLEASE DO NOT READ, SELECT ALL THAT APPLY)<br><br>(NE LISEZ PAS S'IL VOUS PLAIT, SELECTIONNER TOUX CEUX QUI S'APPLIQUENT)	checkbox<br>1 | dss_r2___1 | Routine antenatal visits to clinics/hospital**Visites de routine prénatales dans les cliniques/hôpitaux<br>2 | dss_r2___2 | Clinic visits for pregnancy-related complication or concern**Visites cliniques pour complication ou inquiétude liée a la grossesse<br>3 | dss_r2___3 | Delivery at clinic/hospital**Accoucheme[illegible] a la clinique/Hôpital<br>4 | dss_r2___4 | C-section**C-section<br>5 | dss_r2___5 | Clinic visit for any illness not related to the pregnancy**Visites cliniques pour toutes maladies non lié[illegible] à la grossesse<br>6 | dss_r2___6 | Did not get medications**N'a pas reçu de médicaments<br>7 | dss_r2___7 | Routine postnatal visit at clinic/hospital**Visite de routine postnatale clinique/Hôpital<br>8 | dss_r2___8 | Clinic visits for postnatal concern or complications**Visites cliniques pour des soucis ou des complications postnatale[illegible]<br>88 | dss_r2___88 | Other**Autre<br>98 | dss_r2___98 | Don't know**Ne sait pas<br>99 | dss_r2___99 | Refused to respond**Refus d[illegible] répondre
	60	[dss_r2other]<br><br>Show the field ONLY if:<br>[dss_r2(88)] = '1'	Other, specify<br><br>Autre, specifier	text
	61	[dss_r3]<br><br>Show the field ONLY if:<br>[dss_r] = '1'	R3. Since mid-March, was there a time you needed medical care during the pregnancy but did not receive it?<br><br>R3. Depuis mi-Mars, y a-t-il eu un moment ou vous avez eu besoin de soins médicaux pendant la grossesse, mais que vous n'avez pas reçu?	radio<br>1 | Yes**Oui<br>2 | No**Non<br>98 | Don't know**Ne sait pas<br>99 | Refused to respond**Refus de repondre
	62	[dss_r3a]<br><br>Show the field ONLY if:<br>[dss_r3]='1'	R3a. During this time, how many medical care or clinical visits were missed?<br><br>R3a. Pendant cette période, combien de soins médicaux ou visites cliniques avez-vous manquez?	text
	63	[dss_r4]<br><br>Show the field ONLY if:	R4. What kind of healthcare was needed but not received since mid-March	checkbox<br>2 | dss_r4___2 | Clinic visits for pregnancy-related complication or

		[dss_r3] = '1'	R4. Quel type de soins de santé était-il nécessaire (exige) mais n' pas été reçu depuis mi-Mars?<br><br>(PLEASE DO NOT READ, SELECT ALL THAT APPLY)<br><br>(NE PAS LIRE, SELECTIONNER TOUS CEUX QUI S'APPLIQUENT)	concern**Visites cliniques pour complication ou inquiétude liée a la grossesse<br>3 dss_r4___3 Delivery at clinic/hospital**Accouchemen a la clinique/Hôpital<br>4 dss_r4___4 C-section**C-section<br>5 dss_r4___5 Clinic visit for any illness not related to the pregnancy**Visites cliniques pour toutes maladies non liée à la grossesse<br>6 dss_r4___6 Did not get medications**N'a pas reçu de médicaments<br>7 dss_r4___7 Routine postnatal visit at clinic/hospital**Visite de routine postnatale clinique/Hôpital<br>8 dss_r4___8 Clinic visits for postnatal concern or complications**Visites cliniques pour des soucis ou des complications postnatales<br>88 dss_r4___88 Other**Autre<br>98 dss_r4___98 Don't know**Ne sait pas<br>99 dss_r4___99 Refused to respond**Refus de répondre
	64	[dss_r4other]<br><br>Show the field ONLY if: [dss_r4(88)] = '1'	Other, specify<br><br>Autre, specifier	text
	65	[dss_r5]<br><br>Show the field ONLY if: [dss_r3] = '1'	R5. Why was this care not received?<br><br>R5. Pourquoi ces soins n'ont pas été reçus?<br><br>(PLEASE DO NOT READ, SELECT ALL THAT APPLY)<br><br>(NE LISEZ PAS S'IL VOUS PLAIT, SELECTIONNER TOUS CEUX QUI S'APPLIQUENT)	checkbox<br>1 dss_r5___1 Clinic closed**Fermeture de la clinique<br>2 dss_r5___2 Out of vaccines or medications**A court de vaccin ou de médicaments<br>3 dss_r5___3 Did not get transportation**Faute de moyen de transport<br>4 dss_r5___4 Lockdown**Confinement<br>5 dss_r5___5 Scared to go**Peur d'y aller<br>88 dss_r5___88 Other**Autre<br>98 dss_r5___98 Don't know**Ne sait pas<br>99 dss_r5___99 Refused to respond**Refus de répondre
	66	[dss_r5other]<br><br>Show the field ONLY if: [dss_r5(88)] = '1'	Other, specify<br><br>Autre, specifier	text

	67	[dss_endtime]	Section Header: *THE SURVEY HAS COMPLETED. L'ENQUETE EST TERMINEE. INTERVIEWER SAY: We have completed the survey. Thank you very much for your participation, it contributes a lot to our study. We do appreciate your participation. L'ENQUETEUR DIT: Nous avons terminé l'enquéte.Merci beaucoup pour votre participation, cela contribue beaucoup à notre étude. Nous apprécions votre participation.*<br>Interview End Time:<br><br>HEURE DE FIN DE L'INTERVIEW :	text (time)
	68	[harmonized_covid19_impact_questions_for_champs_dss_complete]	Section Header: *Form Status*<br>Complete?	dropdown<br>0 Incomplete<br>1 Unverified<br>2 Complete

RESPONDENT NUMBER: __ __ __ __ __ __

Harmonized COVID-19 Impact Questions for the CHAMPS HDSS Network

Household DSS ID: ____________ Individual DSS ID: ____________

Interviewer's name: _________________ Interviewer's code: __ __ __

Interview date: __ __/__ __/__ __ __ __

Interview start time: ___________________

INTERVIEWER READ THE FOLLOWING INFORMATION:

(ADD HERE STATEMENT OF THE PURPOSE OF THE STUDY, USE OF DATA, CONFIDENTIALITY, SPONSORS, ETC. AS APPROPRIATE)

INSTRUCTION FOR INTERVIEWER

1. INSTRUCTIONS HAVE BEEN PROVIDED IN CAPITAL LETTERS. DO NOT READ THESE INSTRUCTIONS TO RESPONDENTS.
2. READ ALL QUESTIONS AS WRITTEN. DO NOT READ RESPONSES UNLESS INSTRUCTIONS EXPLICITLY STATE TO DO SO.
3. IF QUESTION INSTRUCTIONS STATE TO READ RESPONSE, DO NOT READ "DON'T KNOW" OR "REFUSED". CIRCLE THESE ONLY IF THEY ARE THE ANSWER GIVEN BY THE RESPONDENTS.
4. INDICATE THE RESPONSE THAT CORRESPONDS TO THE RESPONDENT'S ANSWER.
5. FOR OPEN QUESTION, ENTER THE RESPONDENT'S ANSWER ON THE SPACE PROVIDED.

RESPONDENT NUMBER: __ __ __ __ __ __

Household Demographics

INTERVIEWER TO PARTICIPANT: I would like to ask **the head of the household or their spouse** some questions about your household.

D	Question	Response	Skip
D1	**What is your relationship with the head of household?**<br>(CIRCLE ONE RESPONSE)<br>(THE RESPONDENT SHOULD BE EITHER THE HEAD OF HOUSEHOLD OR THEIR SPOUSE, BUT ADDITIONAL OPTIONS ARE PROVIDED)	Household Head......1<br>Spouse of Household Head......2<br>Son......3<br>Daughter......4<br>Adopted son......5<br>Adopted daughter......6<br>Nephew......7<br>Niece......8<br>Grandson......9<br>Granddaughter......10<br>Father of Household Head......11<br>Mother of Household Head......12<br>Brother of Household Head......13<br>Sister of Household Head......14<br>Unrelated......15<br>Other......88<br>Don't know......98<br>Refused to respond......99	
D2	**What is the current age of the head of household?**<br>(NUMBER OF YEARS)	____________	
D3	**What is the sex of the head of household?**<br>(CIRCLE ONE RESPONSE)	Male......1<br>Female......2<br>Don't know......98<br>Refused to respond......99	

RESPONDENT NUMBER: __ __ __ __ __ __

D4	**What is the current marital status of the head of household?**<br>(CIRCLE ONE RESPONSE)	Married/Cohabitating....1<br>Divorced....2<br>Single/Never married....3<br>Widow/Widower....4<br>Separated....5<br>Other....88<br>Don't know....98<br>Refused to respond....99	
D5	**How many years of education has the head of household completed?**<br>(NUMBER OF YEARS, OR CIRCLE ONE RESPONSE)	________<br>Don't know....98<br>Refused to respond....99	
D6	**Is the head of household currently employed?**<br>(CIRCLE ONE RESPONSE)	Yes....1<br>No....2<br>Don't know....98<br>Refused to respond....99	
D7	**What is the head of household's type of occupation?**<br>(CIRCLE ONE RESPONSE)	Not working....1<br>Professional/Technical/Managerial....2<br>Clerical....3<br>Sales....4<br>Services....5<br>Agricultural – employee....6<br>Skilled manual labor....7<br>Unskilled manual labor....8<br>Other....88<br>Don't know....98<br>Refused to respond....99	
	**Other, specify**	________	

D8	**What is the religion of the head of household?**<br>(CIRCLE ONE RESPONSE)	Islam....1<br>Christian....2<br>Traditional....3<br>Atheist....4<br>Other....88<br>Don't know....98<br>Refused to respond....99	
	**Other, specify**	______________	
D9	**What is the ethnicity of the head of household?**<br>(CIRCLE ONE RESPONSE)	Bambara....1<br>Fula....2<br>Soninke....3<br>Senufo....4<br>Malinke....5<br>Dogon....6<br>Songhai....7<br>Tuareg....8<br>Bobo....9<br>Other....88<br>Don't know....98<br>Refused to respond....99	
	**Other, specify**	______________	
D10	**How many people are in your household?**<br>(TOTAL COUNT OF HOUSEHOLD MEMBERS)	______________<br>Don't know....98<br>Refused to respond....99	
D11	**How many children under the age of 5 are in the household?**	______________<br>Don't know....98<br>Refused to respond....99	

RESPONDENT NUMBER: __ __ __ __ __ __

D12	**How many individuals are 60 years old or older in the household?**	______________<br>Don't know........98<br>Refused to respond........99	
D13	**What is the average total monthly income of your household?**<br>(REPORT IN WHOLE UNITS OF LOCAL CURRENCY)	______________<br>Don't know........98<br>Refused to respond........99	

END OF SECTION (D). GO TO SECTION (V) ON PAGE 6

RESPONDENT NUMBER: __ __ __ __ __ __

Knowledge Regarding the Spread of COVID-19

INTERVIEWER TO PARTICIPANT: I would like to ask **the head of the household or their spouse** some questions about coronavirus or COVID-19

V	Question	Response	Skip
V1	**Have you heard about the coronavirus/ COVID-19?** (CIRCLE ONE RESPONSE)	Yes....1<br>No....2<br>Don't know....98<br>Refused to respond....99	 ->P<br>->P<br>->P
V2	**To your knowledge, which of the following measures can you adopt to reduce the risk of contracting coronavirus/COVID-19?**		

(PLEASE DO NOT READ, CHECK MULTIPLE RESPONSES THAT APPLY, OR WRITE RESPONSE FOR 88)

	No (00)	Yes (01)	Don't know (98)	Refused to Respond (99)
1. Handwashing	☐	☐	☐	☐
2. Sanitizer use	☐	☐	☐	☐
3. Avoid handshake/physical greeting	☐	☐	☐	☐
4. Mask use	☐	☐	☐	☐
5. Gloves use	☐	☐	☐	☐
6. Avoid travel	☐	☐	☐	☐
7. Staying at home	☐	☐	☐	☐
8. Avoid going out unless necessary	☐	☐	☐	☐
9. Avoid crowded places	☐	☐	☐	☐
10. Keep ~2 meters space between yourself and others	☐	☐	☐	☐
11. Other, specify (88)	______________			

RESPONDENT NUMBER: __ __ __ __ __ __

V3	**What steps has your community/government taken to curb the spread of the coronavirus in your area?**<br><br>(PLEASE READ ALOUD, SELECT ALL THAT APPLY)	None....0<br>Advised citizens to stay at home....1<br>Advised to avoid gatherings....2<br>Restricted travel within country/area....3<br>Restricted international travel....4<br>Closure of schools and universities....5<br>Curfew/lockdown....6<br>Closure of non-essential businesses....7<br>Sensitization/public awareness....8<br>Established isolation centers....9<br>Disinfection of public places....10<br>Other....88<br>Don't know....98<br>Refused to respond....99	
	**Other, specify**	______________	
V4	**Were any of your household members tested for COVID-19?**<br><br>(CIRCLE ONE RESPONSE, OR WRITE RESPONSE FOR 88)	Yes....1<br>No....2<br>Don't know....98<br>Refused to respond....99	 ->P<br>->P<br>->P
V5	**Did any of your household members test positive for COVID-19?**<br><br>(CIRCLE ONE RESPONSE, OR WRITE RESPONSE FOR 88)	Yes....1<br>No....2<br>Don't know....98<br>Refused to respond....99	

END OF SECTION (V). GO TO SECTION (P) ON

RESPONDENT NUMBER: __ __ __ __ __ __

Food Availability (P)

INTERVIEWER TO PARTICIPANT: Getting enough food can also be a problem for some households due to coronavirus or COVID-19. I would like to ask you some questions about food availability in your household.

P	Question	Response	Skip
P1a	**Before mid-March 2020, did it ever happen that your household did not have enough food to eat?**<br>(CIRCLE ONE RESPONSE, OR WRITE RESPONSE FOR 88)	Yes........1<br>No........2<br>Don't know........98<br>Refused to respond........99	 ->P2a<br>->P2a<br>->P2a
P1b	**How many times did your household not have enough food to eat?**	______________________<br>Don't know........98<br>Refused to respond........99	
P1c	**Why did your household not have enough to eat or not what you wanted to eat before mid-March 2020?**	Couldn't afford to buy more food........1<br>Couldn't get out to buy food........2<br>Afraid to go or didn't want to go out to buy food........3<br>Couldn't get groceries or meals delivered to me........4<br>Stores didn't have the food I wanted........5<br>Other........88<br>Don't know........98<br>Refused to respond........99	
	**Other, specify**	______________	
P2a	**Since the beginning of the COVID lockdown, has it happened that your household did not have enough food to eat?**	Yes........1<br>No........2<br>Don't know........98<br>Refused to respond........99	->M<br>->M<br>->M

RESPONDENT NUMBER: __ __ __ __ __ __

P2b	**During this time, how many times did your household not have enough food to eat?**	______________________<br>Don't know.......98<br>Refused to respond.......99	
P3	**Why did your household not have enough to eat (or not what you wanted to eat) since mid-March 2020?**<br>(SELECT ALL THAT APPLY)	Couldn't afford to buy more food.......1<br>Couldn't get out to buy food.......2<br>Afraid to go or didn't want to go out to buy food.......3<br>Couldn't get groceries or meals delivered to me.......4<br>Stores didn't have the food I wanted.......5<br>Other.......88<br>Don't know.......98<br>Refused to respond.......99	
	**Other, specify**	______________	

END OF SECTION (P). GO TO SECTION (M) ON PAGE 10

RESPONDENT NUMBER: __ __ __ __ __ __

COVID-19 Related Shocks/Coping (M)

INTERVIEWER TO PARTICIPANT: Now I would like to ask you about events that may have affected your household since mid-March.

M	Question	Response	Skip
M0	**Before mid-March of 2020, were any members of your household engaged in any of the following activities?** (PLEASE READ ALOUD AND SELECT ALL THAT APPLY)	Employment……1<br>Non-farm business operation……2<br>Farming/Agriculture operation……3<br>Don't know……98<br>Refused to respond……99	
M1	**Has your household been affected by any of these events since mid-March?** (PLEASE READ ALOUD AND SELECT ALL THAT APPLY)	Job loss……1<br>Nonfarm business closure……2<br>Disruption of farming……3<br>Disruption of livestock activities……4<br>Disruption of fishing activities……5<br>Increased price of farming or business inputs……6<br>Decreased price of farming or business outputs……7<br>Increased price of major food items consumed……8<br>Illness, injury, or death of any household member……9<br>Not affected by any listed or other major problems/events……10<br>Other……88<br>Don't know……98<br>Refused to respond……99	 <br><br><br><br><br><br><br><br><br>->Q<br>->Q<br>->Q
	**Other, specify**	______________	

CONTINUE SECTION (M) ON PAGE 11

RESPONDENT NUMBER: __ __ __ __ __ __

M2	**How did your household cope with these difficulties encountered since mid-March?**<br><br>(PLEASE DO NOT READ, SELECT ALL THAT APPLY)	Did nothing.....0<br>Sale of assets.....1<br>Engaged in additional income generating activities.....2<br>Assistance from friends & family.....3<br>Borrowed from friends & family.....4<br>Took a Loan.....5<br>Delayed payment obligations.....6<br>Sold harvest in advance.....7<br>Reduced food Consumption.....8<br>Reduced nonfood Consumption.....9<br>Relied on Savings.....10<br>Received assistance from NGO.....11<br>Took advanced payment from employer.....12<br>Received assistance from government.....13<br>Other.....88<br>Don't know.....98<br>Refused to respond.....99	
	**Other, specify**	______________	

END OF SECTION (M). GO TO SECTION (Q) ON PAGE 12

RESPONDENT NUMBER: __ __ __ __ __ __

Under-five Child Healthcare Services (Q)

INTERVIEWER TO PARTICIPANT: Getting adequate health services can be a problem due to coronavirus or COVID-19. I would like to ask some questions about **children under age 5 years** your household.

Q	Question	Response	Skip
Q0	**Since mid-March 2020, were there any children under the age of 5 living in the household?**	Yes....1<br>No....2<br>Don't know....98<br>Refused to respond....99	 ->R<br>->R<br>->R
Q1	**Since mid-March, did any of the children under age 5 attend any healthcare visits?**	Yes....1<br>No....2<br>Don't know....98<br>Refused to respond....99	 ->Q3<br>->Q3<br>->Q3
Q2	**What kind of medical care did the child receive?**<br>(PLEASE DO NOT READ, SELECT ALL THAT APPLY)	Routine follow-up visits for kids....1<br>Routine vaccinations....2<br>Malaria treatment....3<br>HIV treatment....4<br>Clinic visits for any illness....5<br>Services for malnutrition....6<br>Other....88<br>Don't know....98<br>Refused to respond....99	
	**Other, specify**	________________	
Q3	**Since mid-March, was there a time you needed medical care or clinic visit for a baby or child but could not do so?**	Yes....1<br>No....2<br>Don't know....98<br>Refused to respond....99	 ->R<br>->R<br>->R
Q3a	**During this time, how many medical care or**	______________	

	**clinical visits were missed?**		
Q4	**What kind of medical care did the child need but did not receive?** (PLEASE DO NOT READ, SELECT ALL THAT APPLY)	Routine follow-up visits for kids....1<br>Routine vaccinations....2<br>Malaria treatment....3<br>HIV treatment....4<br>Clinic visits for any illness....5<br>Services for malnutrition....6<br>Other....88<br>Don't know....98<br>Refused to respond....99	
	**Other, specify**	_______________	
Q5	**Why did your child not receive healthcare?**<br><br>(PLEASE DO NOT READ, SELECT ALL THAT APPLY)	Clinic closed....1<br>Out of vaccines or medications....2<br>Did not get transportation....3<br>Lockdown....4<br>Scared to go....5<br>Other....88<br>Don't know....98<br>Refused to respond....99	
	**Other, specify**	_______________	

END OF SECTION (Q). GO TO SECTION (R) ON PAGE 14

RESPONDENT NUMBER: __ __ __ __ __ __

Healthcare Services for Pregnant Women (R)

INTERVIEWER TO PARTICIPANT: Getting adequate health services can be a problem due to coronavirus or COVID-19. I would like to ask some questions about **pregnancy health services** during this time.

R	Question	Response	Skip
R0	**Is there a woman in the household who has been pregnant since mid-March 2020?**	Yes........1<br>No........2	 ->End
R1	**Since mid-March, did you attend any pregnancy-related healthcare?**	Yes........1<br>No........2<br>Don't know........98<br>Refused to respond........99	 ->R3<br>->R3<br>->R3
R2	**What kind of healthcare during pregnancy was received since mid-March?**<br><br>(PLEASE DO NOT READ, SELECT ALL THAT APPLY)	Routine antenatal visits to clinics/hospital........1<br>Clinic visits for pregnancy-related complication or concern......2<br>Delivery at clinic/hospital........3<br>C-section........4<br>Clinic visit for any illness not related to the pregnancy........5<br>Did not get medications ....6<br>Routine postnatal visit at clinic/hospital........7<br>Clinic visits for postnatal concern or complications........8<br>Other........88<br>Don't know........98<br>Refused to respond........99	
	**Other, specify**	______________	
R3	**Since mid-March, was there a time you needed medical care during the pregnancy but did not receive it?**	Yes........1<br>No........2<br>Don't know........98<br>Refused to respond........99	 ->End<br>->End<br>->End

RESPONDENT NUMBER: __ __ __ __ __ __

R3a	**During this time, how many medical care or clinical visits were missed?**	______________	
R4	**What kind of healthcare was needed but not received since mid-March**<br><br>(PLEASE DO NOT READ, SELECT ALL THAT APPLY)	Routine antenatal visits to clinics/hospital........1<br>Clinic visits for pregnancy-related complication or concern......2<br>Delivery at clinic/hospital........3<br>C-section........4<br>Clinic visit for any illness not related to the pregnancy........5<br>Did not get medications........6<br>Routine postnatal visit at clinic/hospital........7<br>Clinic visits for postnatal concern or complications........8<br>Other........88<br>Don't know........98<br>Refused to respond........99	
	**Other, specify**	______________	
R5	**Why was this care not received?**<br><br>(PLEASE DO NOT READ, SELECT ALL THAT APPLY)	Clinic closed........1<br>Out of vaccines or medication........2<br>Did not get transportation........3<br>Lockdown........4<br>Scared to go........5<br>Other........88<br>Don't know........98<br>Refused to respond........99	
	**Other, specify**	______________	

SURVEY COMPLETED

RESPONDENT NUMBER: __ __ __ __ __ __

INTERVIEW END TIME: __ __: __ __ AM / PM

THE SURVEY HAS COMPLETED.

INTERVIEWER SAY:

We have completed the survey. Thank you very much for your participation, it contributes a lot to our study. We do appreciate your participation.
